# Optimal schedule of bacillus calmette-guerin for non-muscle-invasive bladder cancer: a meta-analysis of comparative studies

**DOI:** 10.1186/1471-2407-13-332

**Published:** 2013-07-05

**Authors:** Shimiao Zhu, Yang Tang, Kai Li, Zhiqun Shang, Ning Jiang, Xuewu Nian, Libin Sun, Yuanjie Niu

**Affiliations:** 1 Department of Urology, Tianjin Institute of Urology, 2nd Hospital of Tianjin Medical University, Pingjiang Road 23, Tianjin, People’s Republic of China; 2 Department of Urology, Tianjin Third Central Hospital, Tianjin, People’s Republic of China

**Keywords:** Bacillus Calmette-Guérin, Non-muscle-invasive bladder cancer, Maintenance therapy, Low-dose, Combination therapy, Prognosis

## Abstract

**Background:**

To explore the necessity of maintenance, efficacy of low-dose and superiority of various combination therapies of Bacillus Calmette-Guérin (BCG) in treatment of superficial bladder cancer (BCa).

**Methods:**

Comprehensive searches of electronic databases (PubMed, Embase, and the Cochrane Library) were performed, then a systematic review and cumulative meta-analysis of 21 randomized controlled trials (RCTs) and 9 retrospective comparative studies were carried out according to predefined inclusion criteria.

**Results:**

Significantly better recurrence-free survivals (RFS) were observed respectively in patients who received BCG maintenance, standard-dose and BCG plus epirubicin therapy comparing to those received induction, low-dose and BCG alone. BCG maintenance therapy was also associated with significantly better progression-free survival (PFS), but there were more incidences of adverse events. Pooled results showed no remarkable advantage of BCG combined with Mitomycin C or with interferon α-2b in improving oncologic outcomes. Sensitivity-analyses stratified by study-design and tumor stage led to very similar overall results and often to a decrease of the between-study heterogeneity. Our data confirmed that non-RCT only affected strength rather than direction of the overall results.

**Conclusions:**

All patients with superficial BCa should be encouraged to accept BCG maintenance therapy with standard-dose if well tolerated. Patients can benefit from BCG combined with epirubicin but not from BCG combined with Mitomycin C or interferon α-2b.

## Background

More than 30 years ago, intravesical Bacillus Calmette-Guérin (BCG) was first proposed by Morales [1]. Since then, BCG therapy has been demonstrated to be the most effective treatment in the prevention of the recurrence and progression of superficial bladder cancer (BCa), especially for high-risk non-muscle-invasive bladder cancer (NMIBC) [2]. Despite its well-recognized efficacy, many questions remain suspended and among them, the following ones should be noted: 1) the necessity of maintenance BCG therapy; 2) the efficacy of low-dose BCG; and 3) the superiority of combination therapy of BCG.

Previous studies showed that only maintenance BCG could benefit patients in reducing tumor progression [3,4]. However, the results were from studies comparing maintenance BCG with Mitomycin C (MMC) other than BCG induction, and high-level direct evidence supporting maintenance therapy was still absent. The maintenance BCG has been compromised because of serious side-effects (e.g., BCG sepsis and BCG-induced cystitis). Then, low-dose BCG [5-7], recognized to be accompanying with reduced side effects, was introduced. Higher recurrence and progression rates were observed in low-dose group comparing with standard-dose group [5-7], nevertheless, the wide confidential intervals (CI) of individual studies made us failed to detect significant difference between the two groups. So, it’s necessary for us to perform meta-analyses to systematically evaluate the necessity of maintenance BCG and the efficacy of low-dose BCG therapy.

Additionally to optimize the BCG therapy schedules, efficacy of BCG combination therapies (e.g. BCG plus MMC, BCG plus epirubicin and BCG plus interferon α-2b (IFN-α2b)) were also evaluated in this meta-analysis. Many studies had been addressed to improve BCG efficacy by combining with other remedies [8], however, no consistent conclusion was obtained. Thus, the systematic syntheses were addressed to explore the optimal schedule and dose of BCG prescription for NMIBC.

## Methods

### Search strategy and study selection

This meta-analysis was conducted according to the Preferred Reporting Items for Systematic Reviews and Meta-Analyses (PRISMA) [9]. A systematic search of Medline, Embase, and the Cochrane Library was performed using all possible combinations of the following keywords: 1) ‘Low-Dose’ or ‘Low doses’ or ‘maintenance instillation’ or ‘maintenance’ or ‘Mitomycin C’ or ‘MMC’ or ‘Epirubicin’ or ‘interferon’ or ‘IFN’ or ‘combination therapy’ *and* ‘BCG’ or ‘Bacillus Calmette-Guérin’ and ‘bladder cancer’ or ‘Transitional cell carcinoma of bladder’ or ‘urothelial carcinoma of bladder’; 2) and ‘BCG’ or ‘Bacillus Calmette-Guérin’ *and* ‘bladder cancer’ or ‘Transitional cell carcinoma of bladder’ or ‘urothelial carcinoma of bladder’. To perform an extensive search, no language, publication year, or other limits were used. The last quest was updated on March 7, 2013. Reference lists of relevant reviews were hand-searched to identify additional studies.

Eligible studies had to meet the following inclusion criteria: (1) The diagnosis of BCa had to be confirmed pathologically; (2) All patients should be confirmed as NMIBC; (3) Included studies had to provide comparative data; and (4) Only the most recent trials with the greatest number of patients was chosen when overlapped subjects were selected in more than one study.

### Data extraction and quality assessment

Informations were carefully extracted from all eligible publications independently by two authors, and all disagreements were resolved by the third reviewer (Niu) until consensus was achieved on all items. The following data were considered in eligible studies: author name, year and country of the trials, numbers of case and control subjects, age, duration of follow-up, Treatment schedules and doses of medicines, Hazard Ratios (HRs) or Risk Ratios (RRs) and corresponding 95% CIs of each comparisons.

The methodological quality of RCT was assessed by the Jadad scale [10], considering that a high quality RCT should get more than 3 points. The retrospective studies were assessed by the Newcastle-Ottawa scale [11]. Observational studies achieving six or more stars were considered to be of high quality. Assessments were addressed independently by two authors and the disagreements between authors are resolved by discussion. Additionally, all included studies were also evaluated according to the level of evidence (LOE) stated by Phillips et al. [12].

### Statistical analysis

The recurrence-free survival (RFS) and Progression-free survival (PFS) were evaluated to assess the effects of various treatment schedules and doses. The HRs or Risk Differences (RDs) were used to compare all dichotomous variables. HRs were estimated by different methods depending on the data provided in the publication. The simplest method was to collect reported HRs and their 95% CIs in texts. If those data were not available, we looked for the total number of events, the number of patients at risk in each group and the logrank statistic or its P value allowing calculation of an approximation of the HR estimate. If data were only available in the form of survival curve (SC), we extracted from the survival rates at some specified times so as to estimate the HR value and its variance, assuming that the rate of patients censored was constant during the study follow-up [13]. If the data mentioned above were not available, risk ratio should be considered as the last option. To avoid potential publication bias caused by cherry-picking, comparative studies were identified no matter of study design. Then, sensitivity analysis stratified by study-design was conducted to decrease potentially introduced bias by observational studies. In addition, sensitivity analysis stratified by tumor stage was addressed to narrow the population that was suitable for indicated treatment.

Statistical heterogeneity between trials included in the meta-analysis was assessed using Cochrane’s Q statistic [14] . And the inconsistency was quantified by *I*^2^ statistic (100%×[(Q-df)/Q]), higher value denoting greater degree of heterogeneity [15]. Fixed-effects model was used when heterogeneity was not observed; otherwise, random-effects model was used. Fixed-effects model, using the Mantel–Haenszel method, assumed that studies were sampled from populations with the same effect size; whereas the random-effects model, using the Der-Simonian and Laird method, considered that studies were taken from populations with different effect sizes.

Publication bias was evaluated using Begg adjusted rank correlation test and Egger linear regression test. All statistical analyses were conducted using STATA (version 11.0; College Station, Texas) and Review Manage (version 5.1; The Cochrane Collaboration, Oxford). Two-tailed P value of less than .05 was considered statistically significant.

## Results

### Literature search and characteristics of the included studies

**Figure 1:**
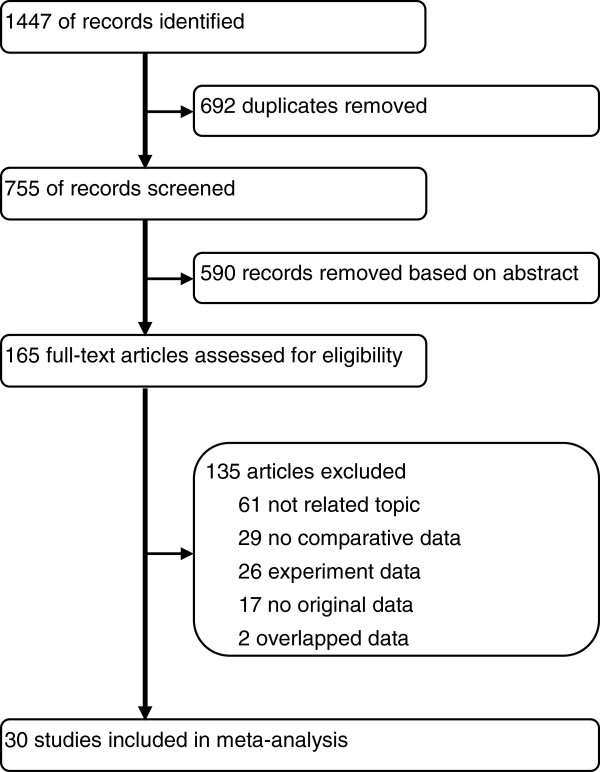
Flowchart of selecting process for meta-analysis.

After removing 692 duplicates, we screened 755 potentially relevant articles. The final number of papers included in the meta-analysis was 30, list of studies excluded and reasons were shown in Figure 1. Of these, nine were identified to explore the necessity of BCG maintenance [16-24], seven investigate the efficacy of low-dose BCG [5-7,25-28], and the remainders address the effects of BCG combination therapy [29-42]. There were 21 randomized-controlled trails (RCTs), eight retrospective studies and one case-series study. Search of the references listed in reviews did not yield any further available studies. Agreement between the two reviewers was 96% for study selection and 96% for quality assessment of trials.

The characteristics of included studies were shown in Tables 1, 2 and 3. Among the RCTs, there were 5 high quality studies (level of evidence: 2b) [6,22,24,33,38]. The severe side-effects that can be easily recognized by both patients and researchers made most of studies failed to use double-blind method, which should be responsible for the low-quality of included RCTs. Only 1 retrospective studies which didn’t adopt appropriate protocol for treatment assignment and used historical series as controls were recognized as low quality case-series study (level of evidence: 4) [42]. The results of quality assessment were listed in Additional file 1: Table S2 and Table S3.

**Table 1:** Characteristics of studies investigating the effects of maintenance of BCG

**First author, yr**	**Design, LOE**	**No. of Case/Control**	Age Case/Control*	**Disease category**	Follow-up, mo Case/Control*	**Strain**	**Treatment dose (mg) Maintenance/Induction**
Badalament 1987 [23]	RCT, 1b	47/46	62.0/63.5	Superficial BCa	22(3–44)	Pasteur	120/120
Koga 2010 [18]	RCT, 1b	24/27	NA	High-risk NMIBC	26.5/28.7	Tokyo	80/80
Hinotsu 2011 [16]	RCT, 1b	41/42	NA	Recurrent or multiple NMIBC	24	Connaught	80/40
Lamm 2000 [22]	RCT, 1b	192/192	66.8/67.0	Recurrent Ta, T1 and CIS	119.1/120.3	Connaught	80/40
Palou 2001 [21]	RCT, 1b	65/61	65.0/63.0	CIS and/or high grade BCa	77.8(7–120)	Connaught	81/81
Hudson 1987 [24]	RCT, 1b	21/21	60.0/62.0	Superficial BCa	16.1±1.4	Pasteur	120/120
Andius 2004 [20]	R, 2b	52/80	74(31–96)	NMIBC without CIS	44(4–155)	Danish/OncoTice	NA
Okamura 2011 [17]	R, 2b	48/27	64.0/68.0	NMIBC without CIS	102/66	Tokyo 172	80/80
Decobert 2008 [19]	R, 2b	31/40	64.3/69.5	High-risk NMIBC	31	Pacis	120/120

*LOE* Level of evidence, *RCT* Randomized-controlled trial, *R* Retrospective, *NMIBC* Non-muscle-invasive bladder cancer, *BCa* Bladder cancer, *CIS* Carcinoma in suit, *NA* Not available, *Case/Control*, BCG maintenance/Induction.

* Mean or median.

**Table 2:** Characteristics of studies investigating the effects of low-dose BCG

**First author, yr**	**Design, LOE**	**No. of Case/Control**	Age Case/Control*	**Disease category**	Follow-up, mo Case/Control*	**Strain**	**Treatment dose (mg) Case/Control**	Maintenance** Case/Control
Ojea 2007 [25]	RCT, 1b	139/142	64.9/65.1	Intermediate-Risk NMIBC	61.2/57.3	Connaught	13.5/27	Yes/Yes
Yalcinkaya 1998 [7]	RCT, 1b	25/25	56.3/55.3	Superficial BCa	26.8/31.6	Connaught	54/81	No/No
Kumar 2002 [6]	RCT, 2b	13/13	55.9/56.7	NMIBC without CIS	24(12–30)	Danish 1331	40/120	No/No
Oddens 2013 [28]	RCT, 1b	678/677	68.0/67.0	Intermediate and high risk NMIBC	85.2	OncoTice	27/81	Yes/Yes
Takashi 1995 [27]	R, 2b	37/37	68.1/61.4	Superficial BCa	32	Tokyo 172	40/80	No/No
Yoneyama 2008 [5]	R, 2b	65/85	68.3/65.5	NMIBC without CIS	42.2/90.7	Tokyo 172	40/80	No/No
Irie 2003 [26]	R, 2b	41/39	61.6/62.2	Superficial BCa	27.5/20.0	Tokyo 172	40/80	No/No

*LOE* Level of evidence, *RCT* Randomized-controlled trial, *R* Retrospective, *NMIBC* Non-muscle-invasive bladder cancer, *BCa* Bladder cancer, *Case/Control*, Low-dose/Standard-dose BCG.

* Mean or median.

** Maintenance is defined as any instillation beyond the induction course.

**Table 3:** Characteristics of studies investigating Combination BCG therapies

**First author, yr**	**Design, LOE**	**No. of**	Age*	**Disease category**	**Follow-up, mo**	**Strain**	**Treatment schedules**	
		**Case/Control**	**Case/Control**		Case/Control*		**Case**	**Control**
**BCG plus mitomycin C vs. BCG alone (Case vs. Control)**								
Kaasinen 2003 [34]	RCT, 1b	159/145	71.0/69.9	CIS	56.3(1.9-97.3)	Connaught	6 weekly BCG plus alternating BCG and MMC monthly	6 weekly BCG plus monthly BCG
Stasi 2006 [33]	RCT, 1b	107/105	66.0/67.0	High-risk NMIBC	91/84	Connaught	2 weekly BCG and 1 week MMC as one cycle for three cycles**	6 weekly BCG**
Oosterlinck 2011 [30]	RCT, 1b	48/48	68.0/70.0	CIS	56.4	Tice	6 weekly MMC plus 6 weekly BCG**	6 weekly BCG plus 3 weekly rest and BCG**
Gulpinar 2012 [29]	RCT, 1b	25/26	58.2/58.0	Intermediate-Risk NMIBC	41.3/40.9	Tice	Perioperative MMC plus 6 weekly BCG	6 weekly BCG
El Mohsen 2010 [32]	RCT 1b	29/27	47.5/48.1	Superficial BCa	30	NA	Perioperative MMC plus 4 weekly MMC plus monthly BCG for 1 year	6 weekly BCG plus monthly BCG for 1 year
Badalato 2011 [31]	R, 2b	48/212	69.6/69.6	Superficial BCa	33.0/43.6	Connaught	Perioperative MMC plus 6 weekly BCG**	6 weekly BCG **
**BCG plus mitomycin C vs. mitomycin C alone (Case vs. Control)**								
Rintala 1996 [37]	RCT, 1b	95/93	68/69	Rapidly recurring NMIBC	34(1–76)	Pasteur	Alternating courses of MMC and BCG monthly during year 1 and every 3 months during year 2	MMC monthly during year 1 and every 3 months during year 2
Jarvinen 2012 [35]	RCT, 1b	28/40	66/68	CIS	408	Pasteur	Ditto	Ditto
Witjes 1998 [36]	RCT, 1b	90/92	NA	Intermediate-Risk NMIBC	32(2–65)	Tice	4 weekly MMC followed by 6 weekly BCG	10 weekly MMC
**BCG plus Epirubicin vs. BCG alone (Case vs. Control)**								
Cai 2008 [38]	RCT, 1b	80/81	73.9/69.8	High-risk NMIBC	15.3/14.8	OncoTice	Perioperative epirubicin plus delayed BCG	6 weekly BCG and every 3 months for maintenance
Bilen 2000 [40]	RCT, 1b	20/21	57/53	High-risk NMIBC	18(9–24)	Connaught	Sequential instillation of BCG and epirubicin for 8 weeks	6 weekly BCG
Tozawa 2001 [39]	R, 2b	24/50	67.9/65.6	Superficial BCa without CIS	6-36	Tokyo	Epirubicin and BCG were instilled by turns once a week for 12 weeks	6 weekly plus six monthly BCG
**BCG plus Interferon α-2b vs. BCG alone (Case vs. Control)**								
Nepple 2010 [41]	RCT, 1b	346/324	68.4	Superficial BCa	NA	Tice	6 weekly BCG plus IFN**	6 weekly BCG **
Bazarbashi 2000 [42]	C-S, 4	37/18	59/58	Superficial BCa	26.2/23.8	Connaught	Weekly sequential BCG and IFN for 8 weeks	6 weekly BCG

*LOE *Level of evidence, *RCT* Randomized-controlled trial, *R* Retrospective, *C-S* Case-series, *NMIBC* Non-muscle-invasive bladder cancer, *BCa* Bladder cancer, *CIS* Carcinoma in suit, *NA* Not available.

* Mean or median.

** Patients were rendered bladder cancer-free were given maintenance courses, Maintenance is defined as any instillation beyond the induction course.

### Meta-analysis results

#### BCG maintenance vs. induction alone

**Figure 2:**
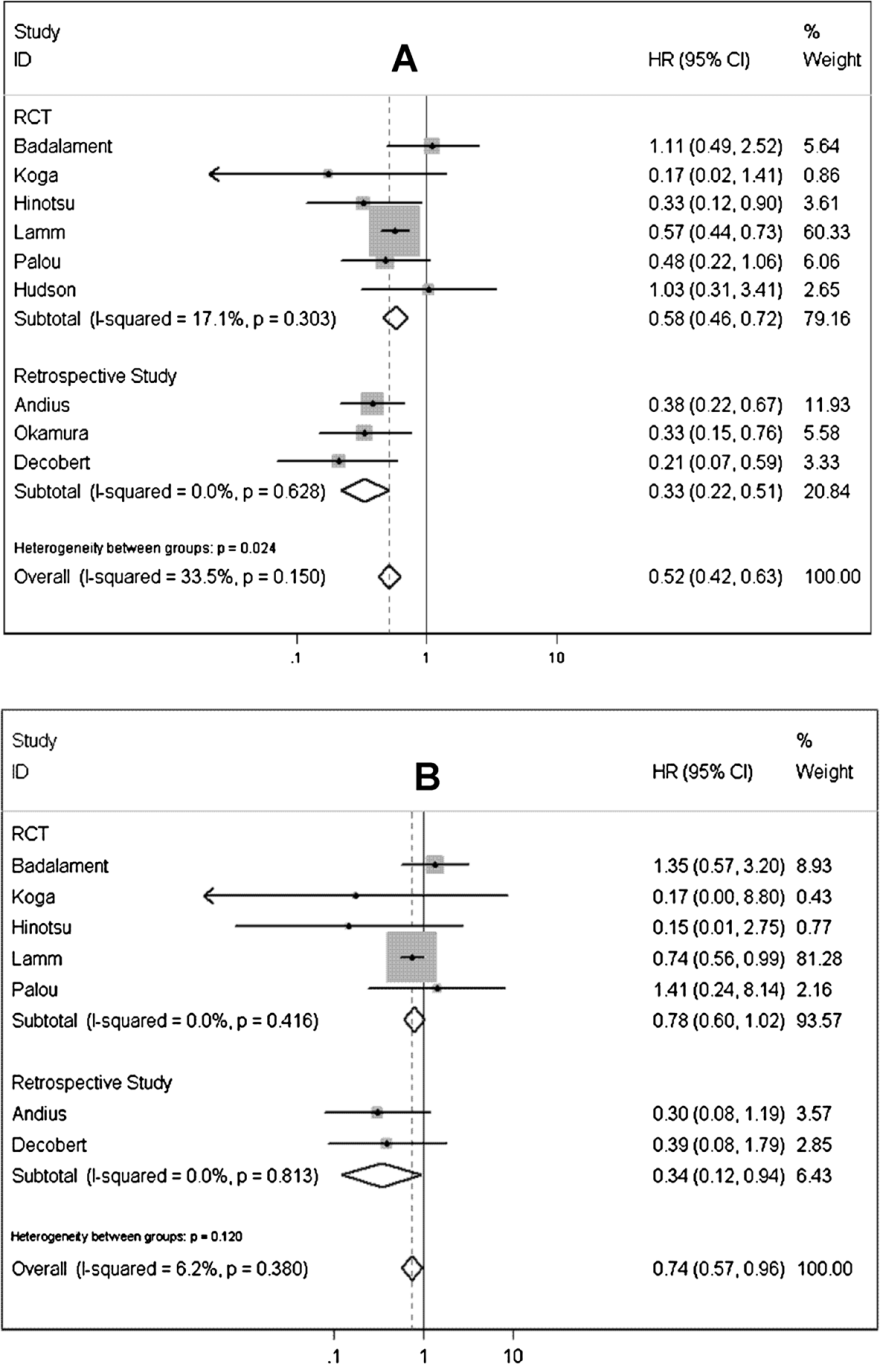
Forest plots of pooled results (A, recurrence-free survival; B, progression-free survival) for the maintenance group and non-maintenance group.

In this meta-analysis of comparative studies, nine studies were identified to investigate the necessity of BCG maintenance therapy [16,18-24]. Pooled results of data that assessed tumor recurrence rate showed that BCG maintenance therapy could significantly improve RFS (HR=0.516; 95% CI 0.425-0.627; *P*<0.0001) (Figure 2A), comparing with BCG induction. Cancer progression rates were investigated in 7 studies, and a notable advantage of BCG maintenance in improving PFS was also observed (HR=0.740; 95% CI 0.572-0.957; *P*=0.022) (Figure 2B). Five studies were available to explore the RFS and PFS in high-risk NMIBC patients underwent BCG maintenance or induction therapy [16,18,19,21,22], significant differences were presented between the two treatment schedules (RFS: HR=0.515, 95% CI 0.411-0.646, *P*<0.0001; PFS: HR=0.722, 95% CI 0.548-0.951, *P*=0.020) (Table 4). No heterogeneity among included studies was significant for the above all analyses (Table 4).

**Table 4:** Pooled results of recurrence-free survival, progression-free survival and publication bias of comparing various BCG treatment schedules and doses

**Measurement**	n*	**Case or case control**	**Heterogeneity**		**Pooled rate / HR**	**Begg’s test ( *P *)**	**Egger’s test ( *P *)**
			***P***	***I***^***2 ***^**(%)**	**(95% CI)**		
**Recurrence-free survival**							
*Superficial bladder cancer*							
Maintenance vs. Induction	9	521/536	0.150	12.0	**0.516 (0.425-0.627)**	0.602	0.353
Low-dose vs. Standard-dose	7	998/1018	0.831	0.00	**1.162 (1.051-1.285)**	1.000	0.126
BCG plus MMC vs. BCG	6	416/563	<0.001	79.1	0.726 (0.490-1.075)	0.452	0.334
BCG plus MMC vs. MMC	3	213/225	0.297	17.7	0.854 (0.663-1.099)	0.296	0.132
BCG plus Epirubicin vs. BCG	3	124/152	0.652	0.00	**0.618 (0.384-0.993)**	1.000	0.538
BCG plus IFN-α2b vs. BCG	2	383/342	0.621	0.00	1.075 (0.859-1.345)	1.000	NA
*High-risk NMIBC*							
Maintenance vs. Induction	5	353/362	0.272	22.4	**0.515 (0.411-0.646)**	0.086	0.032
BCG plus MMC vs. BCG	3	314/298	<0.001	89.8	0.852 (0.449-1.617)	1.000	0.689
BCG plus MMC vs. MMC	2	123/133	0.119	58.7	0.797 (0.477-1.330)	1.000	NA
BCG plus Epirubicin vs. BCG	2	100/102	0.556	0.00	**0.544 (0.302-0.980)**	1.000	NA
**Progression-free survival**							
*Superficial bladder cancer*							
Maintenance vs. Induction	7	452/488	0.380	6.20	**0.740 (0.572-0.957)**	0.764	0.399
Low-dose vs. Standard-dose	6	961/981	0.829	0.00	1.151 (0.853-1.554)	0.452	0.112
BCG plus MMC vs. BCG	3	314/298	0.001	85.3	0.602 (0.181-1.999)	1.000	0.420
BCG plus MMC vs. MMC	3	213/225	0.827	0.00	0.927 (0.482-1.784)	1.000	0.493
BCG plus Epirubicin vs. BCG	2	100/102	0.980	0.00	0.513 (0.132-1.987)	1.000	NA
*High-risk NMIBC*							
Maintenance vs. Induction	5	353/362	0.581	0.00	**0.722 (0.548-0.951)**	0.806	0.311
BCG plus MMC vs. BCG	3	314/298	0.001	85.3	0.602 (0.181-1.999)	1.000	0.420
BCG plus MMC vs. MMC	2	123/133	0.813	0.00	0.829 (0.387-1.773)	1.000	NA
BCG plus Epirubicin vs. BCG	2	100/102	0.980	0.00	0.513 (0.132-1.987)	1.000	NA

*NA* not applicable. Significant datas are emphasized in bold.

* Number of included studies.

#### Low-dose vs. standard-dose BCG

**Figure 3:**
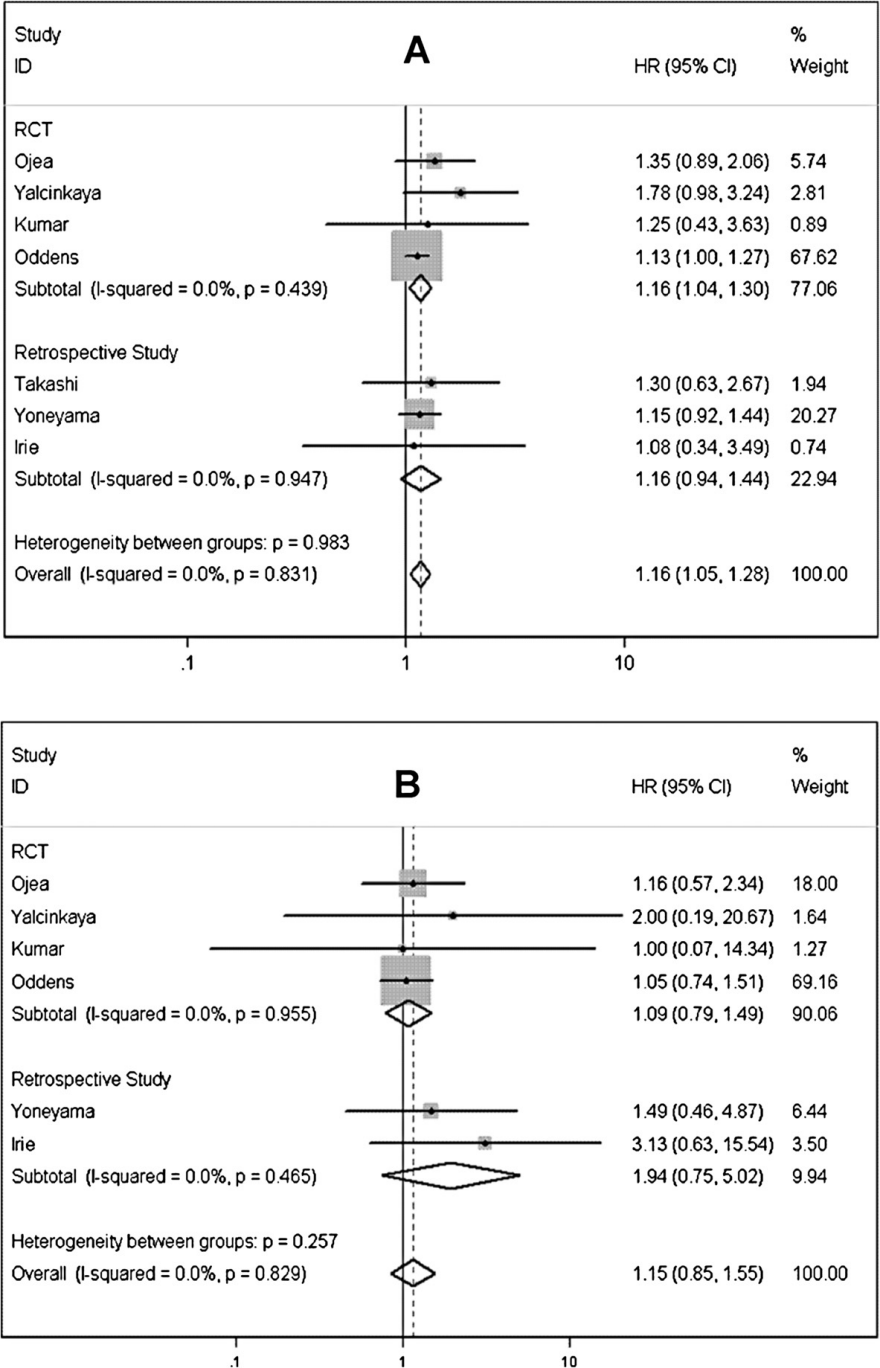
Forest plots of pooled results (A, recurrence-free survival; B, progression-free survival) for the low-dose group and standard-dose group.

In term of RFS, six studies [5-7,25-28] including 998 cases and 1018 controls were identified to comparing low dose BCG to standard doses, cumulated result showed a remarkable difference opposing to low-dose BCG (HR=1.162; 95% CI 1.051-1.285; *P*=0.003) (Figure 3A). Pooling data of 5 studies [5-7,25,26,28] including 1,942 patients that reported tumor progression rate showed the PFS was lower in low-dose BCG than in control group, but this difference was not statistically significant (HR=1.151; 95% CI 0.853-1.554; *P*=0.356) (Figure 3B). Significant heterogeneity was detected in neither analysis above (Table 4).

#### BCG plus mitomycin C vs. BCG alone

**Figure 4:**
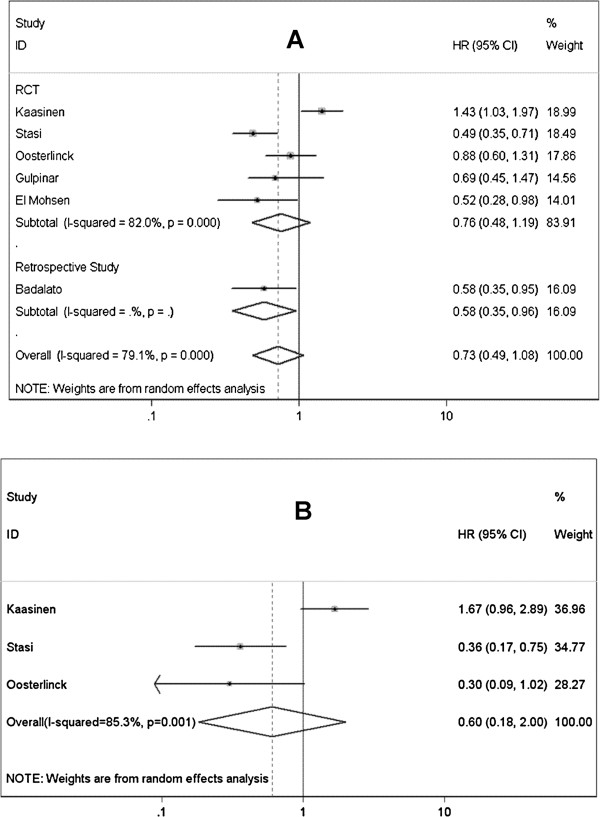
Forest plots of pooled results (A, recurrence-free survival; B, progression-free survival) for the BCG plus Mitomycin C group and BCG alone group.

Six studies involving 979 patients with superficial BCa were identified to investigate the difference between therapy schedules of BCG alone and BCG plus MMC [29-34], synthesized data showed no significantly better RFS in patients accepted combined therapy (HR=0.726; 95% CI 0.490-1.075; *P*=0.110) (Figure 4A). High-risk NMIBC was evaluated in 3 of above 5 studies [30,33,34], also no significant result was observed (HR=0.852; 95% CI 0.449-1.617; *P*=0.624). Tumor progression rate was available in 3 studies, in these studies, only high-risk NMIBC patients were included [30,33,34]. Pooled analysis didn’t show significantly different outcome between BCG plus Mitomycin C and BCG alone (HR=0.602; 95% CI 0.181-1.999; *P*=0.407) (Figure 4B). There were significant heterogeneities in all above 3 analyses, so random-effect models were selected (Table 4).

#### BCG plus mitomycin C vs. mitomycin C alone

**Figure 5:**
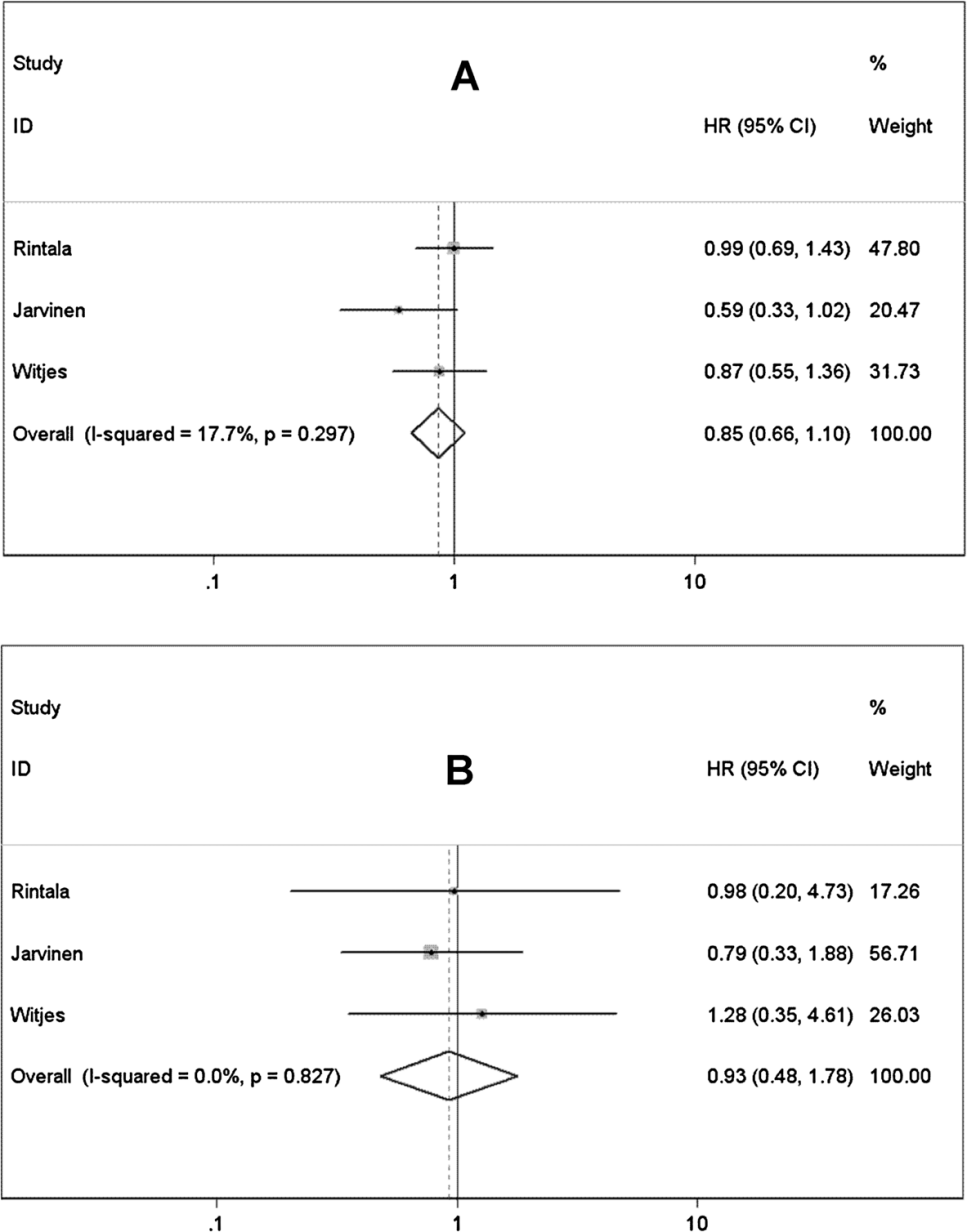
Forest plots of pooled results (A, recurrence-free survival; B, progression-free survival) for the BCG plus Mitomycin C group and Mitomycin C alone group.

Pooled data of 3 RCTs [35-37] didn’t revealed BCG plus MMC significantly improved the RFS or PFS of patients with superficial BCa comparing with MMC alone (RFS: HR=0.854, 95% CI 0.663-1.099, *P*=0.220; PFS: HR=0.927, 95% CI 0.482-1.784, *P*=0.822) (Figure 5A and B). High-risk NMIBC were investigated in 2 studies [35,37], integrated data also failed to detect remarkable advantage of combined therapy in reducing recurrence and progression rates comparing to MMC alone (RFS: HR=0.797, 95% CI 0.477-1.330, *P*=0.385; PFS: HR=0.829, 95% CI 0.387-1.773, *P*=0.628). There is no evidence of between-study heterogeneity in these analyses mentioned above (Table 4).

#### BCG plus epirubicin vs. BCG alone

**Figure 6:**
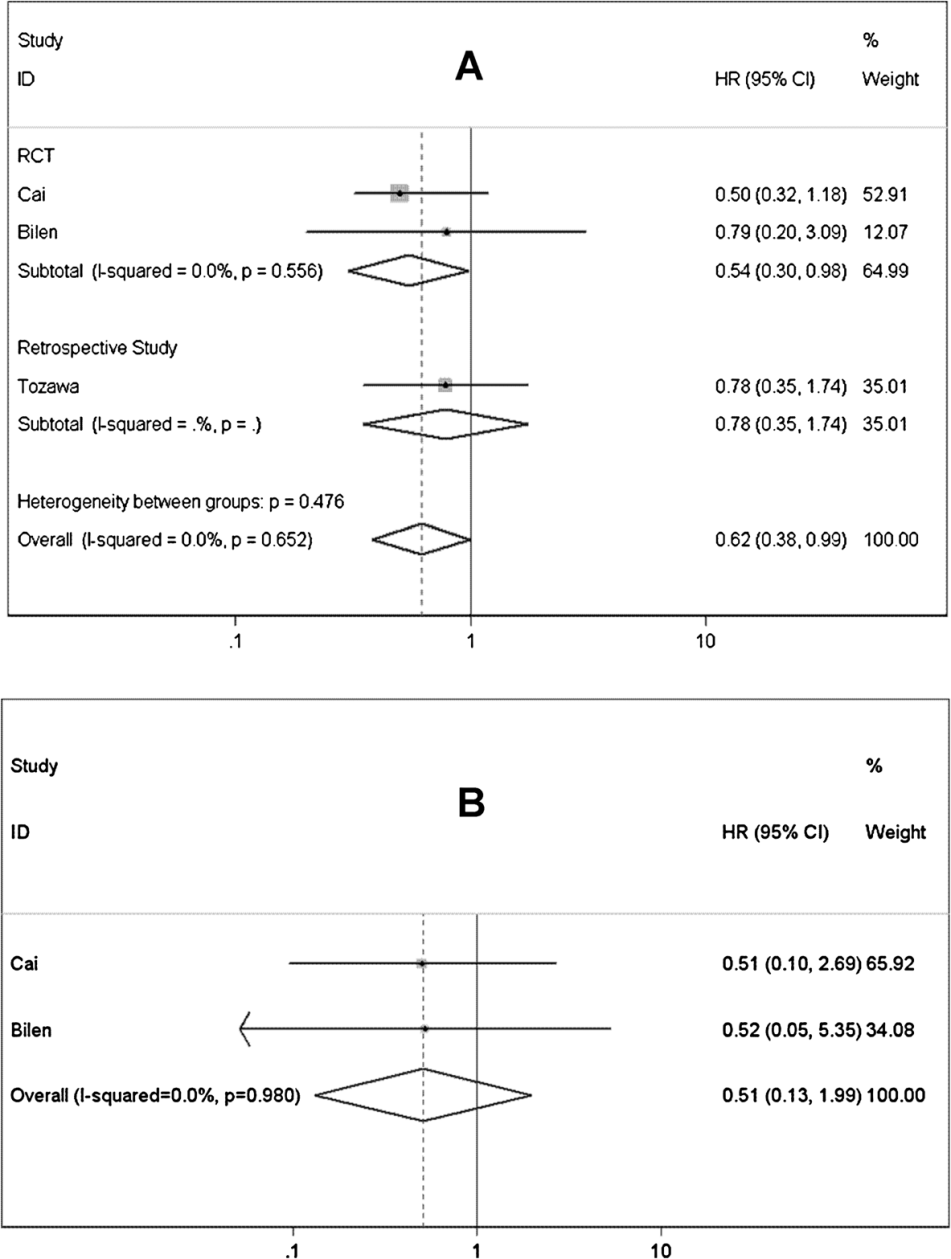
Forest plots of pooled results (A, recurrence-free survival; B, progression-free survival) for the BCG plus Epirubicin group and BCG alone group.

Evidence from 3 studies [38-40] showed that combination of BCG and epirubicin could significantly prevent or delay the recurrence of superficial BCa (HR=0.618; 95% CI 0.384-0.993; *P*=0.047) (Figure 6A). The combination therapy had a more significant effect on preventing high-risk NMIBC from recurrence (HR=0.544; 95% CI 0.302-0.980; *P*=0.043). Progression rate was reported in 2 studies including 202 high-risk NMIBC patients [38,40], better outcome was observed in patients received combined therapy of BCG plus epirubicin, but this difference was not statistically significant (HR=0.513; 95% CI 0.132-1.987; *P*=0.334) (Figure 6B). The homogeneity among included studies was recognized by heterogeneity test (Table 4).

#### BCG plus interferon α-2b vs. BCG alone

**Figure 7:**
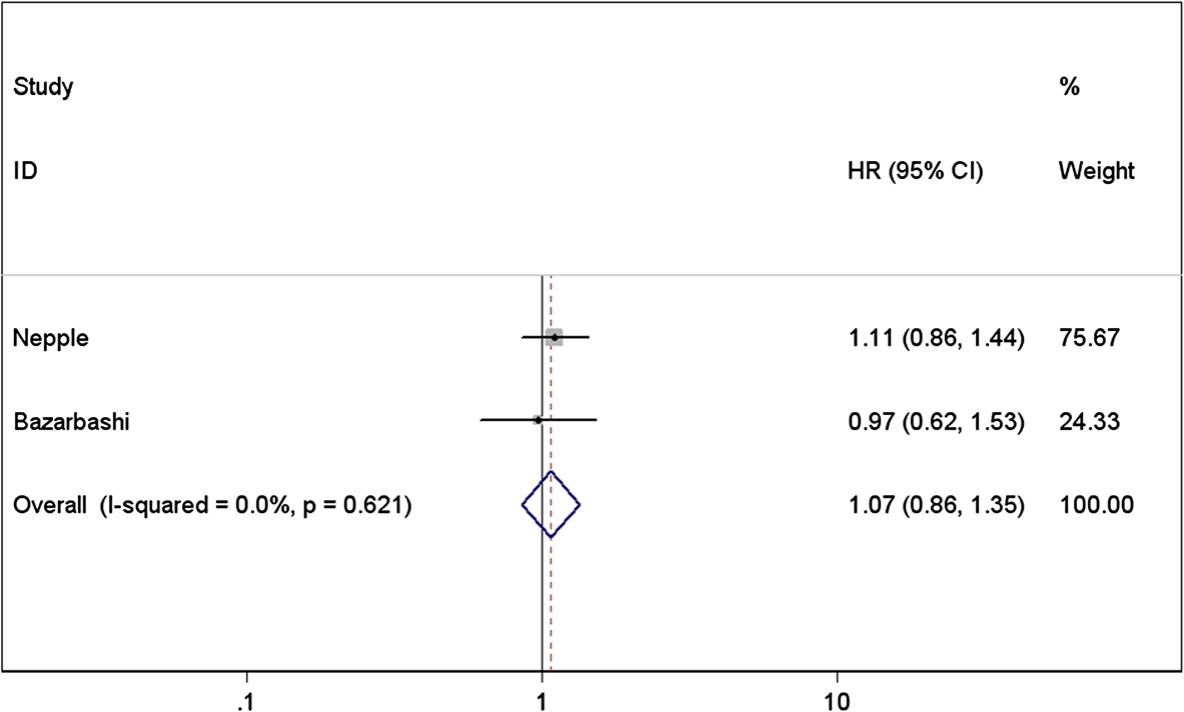
Forest plots of pooled results of recurrence-free survival for the BCG plus Interferon α-2b group and BCG alone group.

Only RFS was discussed for this comparison, no significant difference was found between the two groups (HR=1.075; 95% CI 0.859-1.345; *P*=0.527) (Figure 7), and no evidence of heterogeneity was presented (Table 4).

#### Complications of treatment

One of the most important indicators of complications was the percentage of patients who are unable to complete the treatment course because of side effects. Unfortunately, only therapy schedule of BCG plus MMC could be assessed by cessation rate due to side effects. As shown in Additional file 1: Figure S1, less cessation occurred in group of BCG plus MMC comparing to BCG or MMC alone, but no significant difference was detected (HR=0.553, 95% CI 0.170-1.797, *P*=0.325; HR=0.696, 95% CI 0.334-1.454, *P*=0.335; respectively).

Then the side effects accompanied with therapy were evaluated to oppose treatment efficacy. Numbers of limitation emerged hindering us from evaluating side effects caused by various therapy schedules and doses: 1) descriptors reported in individual articles were of great difference; 2) variety of terms were used to report complications; 3) only simple descriptions of complications were present in the most of included studies. The AUA guidelines panel for NMIBC combined complications into a set of categories [43]: bladder contracture, epididymitis/prostatitis/urethral infections, hematuria, lower urinary tract symptoms (LUTS) (including frequency, urgency, dysuria, etc.), fever/chills/flu symptoms, and systemic infection. According to the guideline panel, side-effects rates were present in a manner of ‘maximal overlap’, since it’s unlikely that each patient had only one symptom. For example, a chilled man usually gets into fever. Maximal overlap indicates that different symptoms within the same category occurred simultaneously in the same patients [43].

Additional file 1: Table S1 listed common complications related to various treatments. The most common local and systemic side effects are LUTS and flu-like symptoms, respectively. Pooled risk differences of side effects that caused by different therapy schedules were showed in Additional file 1: Figure S2. The occurrence of local (e.g. hematuria and LUTS) and systemic (e.g. flu-like symptoms) side effects were both significantly elevated by maintenance therapy. Low-dose BCG only reduced the incidence of LUTS with a cautious significance, while no remarkable difference was present in other comparisons.

#### Sensitivity analysis and publication bias

For sensitivity analysis, we only included RCT. Subgroup data showed that BCG maintenance was no longer significantly prevent patients from getting the chance of progression (HR=0.781; 95% CI 0.598-1.019; *P*=0.068; Figure 2B). However, the obvious trend favoring BCG maintenance could be found still. And there was no significant difference between the results obtained from studies with the two distinct designs (*P*=0.12). No other remarkable change was present in subgroup analyses stratified by study design. For difference between results obtained from trials and those with other study designs, the significance was only noted in Figure 1A (*P*=0.03) Inclusion of the non-randomized studies might inappropriately inflate the power of the analyses, thus the findings on RFS in this analysis should be interpreted with caution. Data from subgroup-analyses confirmed that non-RCT only affected strength rather than direction of the overall results. Another sensitivity analysis stratified by pathologic tumor stage was also conducted, as shown in Table 4, no variation was detected. The Begg’s and Egger’s tests (Table 4) revealed that significant publication bias existed in only 1 (BCG maintenance vs. induction for RFS in high-risk NMIBC group) of all comparisons performed in the present analysis.

## Discussion

In this systematic review, we included 21 RCTs and 9 retrospective studies to evaluate the optimal schedule and dose of BCG for preventing recurrence and progression of superficial BCa. Pooled data revealed that BCG maintenance could significantly improve RFS and PFS in patients with superficial BCa, especially for patients with high-risk NMIBC. Our results were similar to the indirect evidence proved by Bohle et. al [3] and Sylvester et. al [4], which showed that maintenance therapy was associated with better outcomes, but sole induction course didn’t seem to have superiority comparing with MMC. Accompanied with better clinical outcomes, incidence of adverse events was raised when patients receiving maintenance therapy. The potential benefits of BCG maintenance seemed to outweigh the risk of complications, even it was serious, and especially for patients with tumors that carried substantial risk of progression and might ultimate death from bladder cancer. In a high-quality cohort study, Decobert et al. [19] suggested that patients should be encouraged to tolerate at least 3 cycles of maintenance and to continue further instillations if well tolerated. However, serious side-effects caused by long-term BCG may be unworthy for those with low-risk lesions. Andius and Holmang showed that multiple instillation cycles may not be necessary for pTa and lower-grade tumors [20]. Unfortunately, there is no sufficient data to state if low-risk NMIBC could benefit from maintenance therapy. AUA guideline suggested that these risks and benefits should be discussed with the patients [43].

This meta-analysis shows that low-dose BCG may be defective in preventing tumor recurrence, which opposes to previous studies that clarified no significantly different RFS existed between low- and standard-doses BCG for patients with superficial BCa, even for those with high-risk NMIBC (e.g. T1G3) [5,6,26]. In an effort to reduce the potential of complications caused by BCG (e.g. LUTS), pooled results have demonstrated sufficient efficacy using lower dose of BCG. Low-dose BCG can be chosen by patients with low-risk BCa, and then adverse effects can be minimized. Nevertheless, there is no evidence support the hypothesis.

Evidence in a cautious manner showed that sequential intravesical epirubicin could improve BCG efficacy regarding RFS in patients with superficial BCa (e.g. high-risk NMIBC). It was logical to deduce that treatment with sequential chemoimmunotherapy with two different antitumor mechanisms might be more active than monotherapy alone. However, pooled results of BCG combined with MMC diverged from the hypothesis because we did not find significant difference in terms of RFS and PFS between patients who received intravesical MMC plus sequential BCG and those who received only BCG instillations. Significant heterogeneities were observed in this analysis and corresponding subgroup-analysis. Slight difference of treatment schemes existed in the therapy schedules and the different duration of follow-up period among the included studies may be responsible for them. Moreover, the combination use of BCG and MMC wasn’t significantly superior to MMC alone. MMC may cause low efficacy of the combined treatment, because it can repress the immune response while preventing implantation of carcinoma. Basis researches were awaited to conduct the issue.

IFN-α2b is thought to increase the response of Th1 cell caused by BCG instillation through multiple approaches such as inhibiting interleukin-10 and enhance tumor necrosis factor-related apoptosis-inducing ligand release, ultimately lead to the suppression and subsequent destruction of urothelial carcinoma [44,45]. But we failed to observe a major efficacy after giving intravesical IFN-α2b. However, IFN-α2b may be useful for patients with BCG refractory cases, for whom the BCG alone is insufficient to effectively awake the immune response to carcinoma. The final results of a national multicenter study of BCG plus IFN-α2b for treating superficial BCa confirmed the hypothesis [46]. The results showed a 59% and 45% recurrence-free rate of patient naive to BCG and those having BCG failure at a median follow-up of 24 months, respectively.

The overall results, except one, did not change remarkably after subgroup and sensitivity analyses. Publication bias was only observed in one subgroup analysis, which was proved by Egger’s test. Our analysis combined the data from all studies that passed our predefined criteria; therefore, we are confident of the validity of our findings.

However, some inherent limitations of this meta-analysis should also be taken into account when interpreting our data. Firstly, most RCTs included in our analysis were of low-quality, which was caused by obvious side-effects which made it difficult to address double-blind. Another limitation of this study is the publication bias observed in subgroup analysis. This might impact the interpretation of the results because unpublished data may overturn this obtained result. So this result should be interpreted with caution. One reason for the bias may be that only English articles were searched, because other languages such as German were out of our ability. This selection might favor the positive studies that were more often published in English while the negative ones tended to be more often reported in native languages [47]. We attempted to minimize the publication bias by making our literature search as extensive as we could. Moreover, the clinical and pathologic stages of patients that were important to the oncologic prognosis were different in the included trials, which might substantially confound the presented results. Furthermore, slight difference of treatment schemes existed in the same therapy schedules, so the studies with standard schemes and enough follow-up time are expected. Finally, different duration of follow-up period among the included studies also affected the outcomes. To lessen the effect of follow-up period on synthetic results, HRs (time variable was taken into account) were prioritized in included studies.

## Conclusions

Pooled result shows that BCG maintenance is associated with better oncologic outcomes (e.g. RFS and PFS). However, there is a higher incidence of adverse events. Low-dose BCG can’t effectively prevent the recurrence of tumor, though a slightly reduced incidence of LUTS is observed. Compared with single BCG, combination with epirubicin may significantly reduce recurrence but not progression rate, and no more side-effect emerges. For both RFS and PFS, combination BCG with MMC or IFN-α2b is not superior to BCG and MMC alone. Given that the low-quality of the included studies can’t be overcomed, large-volume, well-designed, RCTs with extensive follow-up are needed to confirm and update our findings.

## Abbreviations

BCG: Bacillus calmette-guerin; BCa: Bladder cancer; NMIBC: Non-muscle-invasive bladder cancer; RFS: Recurrence-free survivals; PFS: Progression-free survival; MMC: Mitomycin C; CI: Confidential intervals; IFN-α2b: Interferon α-2b; PRISMA: Preferred reporting items for systematic reviews and meta-analyses; HR: Hazard ratios; RR: Risk ratios; RD: Risk differences; LOE: Level of evidence; SC: Survival curve; RCT: Randomized-controlled trails; LUTS: Lower urinary tract symptoms.

## Competing interests

All authors declared that they have no competing interest.

## Authors’ contributions

YN originated the idea for the paper. YT and HZ carried out the systematic search. ZS, NJ and XN did the data extraction and analysis. SZ drafted the paper with assistance from YN, YT and KL. KL and SZ revised the manuscript according to the suggestions from the editor and reviewers. All authors reviewed the paper critically and have read and approved the manuscript for publication. All authors had full access to all the data in the study and take responsibility for the integrity of the data and the accuracy of the data analysis. YN are the guarantor. All authors have read and approved the final manuscript.

## Pre-publication history

The pre-publication history for this paper can be accessed here:

http://www.biomedcentral.com/1471-2407/13/332/prepub

## Supplementary Material

Additional file 1: Table S1 Side effects of various BCG therapy schedules and doses. **Table S2.** Quality assessment for RCTs using Jadad (5-ponit). **Table S3.** Quality assessment for retrospective study using Newcastle-Ottawa Scale. **Figure S1.** Summary of cessation-free survival for combination of BCG and Mitomycin C (A, BCG plus Mitomycin C vs. BCG alone; B, BCG plus Mitomycin C vs. Mitomycin C alone). **Figure S2.** Summary risk difference of side-effect for various BCG therapy schedules and doses. Click here for file
