# Supplementary material for: Optimal schedule of bacillus calmette-guerin for non-muscle-invasive bladder cancer: a meta-analysis of comparative studies
Source: BMC Cancer. 2013 Jul 5;13:332. doi: 10.1186/1471-2407-13-332 (PMC3722001; doi:10.1186/1471-2407-13-332)

Table S1 Side effects of various BCG therapy schedules and doses

	Bladder Contracture		Epid/Prost/Ure Inf		Hematuria		LUTS		Fever/Chills/Flu symptoms	
	Case*	Control*	Case*	Control*	Case*	Control*	Case*	Control*	Case*	Control*
BCG maintenance vs. Induction (Case vs. Control)										
Hinotsu 2011			1/42	0/42	39/42	30/42	39/42	30/42	18/42	11/42
Decobert 2008					17/31	7/40	17/31	7/40	7/31	2/40
Low-dose vs. Standard-dose BCG										
Yalcinkaya 1998			1/25	2/25	14/25	6/25	19/25	24/25	10/25	12/25
Yoneyama 2008					9/65	21/85	31/65	48/85	12/65	20/85
Irie 2003					3/34	7/30	10/37	20/38	2/35	5/39
BCG plus Epirubicin vs. BCG alone (Case vs. Control)										
Bilen 2000	1/20	0/21			4/20	8/21	7/20	9/21	2/20	3/21
Tozawa 2001	0/24	2/50					13/24	20/50	5/24	7/50
BCG plus Interferon α-2b vs. BCG alone (Case vs. Control)										
Nepple 2010									38/346	16/324

BCG, bacillus Calmette-Guérin; CI, confidence interval; Est Rate, estimated occurrence rate; Epid, epididymitis; Ure Inf, Urethral Infections; G/P, Number of group/Number of patients; Induct, induction; LUTS, lower urinary tract symptoms; Maint, maintenance; MMC, mitomycin C; Prost, prostatitis; TURBT, transurethral resection of bladder tumor

* Event / Total numbers

Table S2 Quality assessment for RCTs using Jadad (5-ponit)

Studies	Randomization	Double blinding	Withdrawals and Dropouts	Total Score
Maintenance vs. Induction				
Badalament 1987	+	-	+	2
Koga 2010	+	-	+	2
Hinotsu 2011	+	-	+	2
Lamm 2000	++	-	+	3
Palou 2001	+	-	+	2
Hudson 1987	++	-	+	3
Low-dose vs. Standard-dose				
Ojea 2007	+	-	+	2
Yalcinkaya 1998	+	-	-	1
Kumar 2002	+	+	+	3
Oddens 2013	+	-	+	2
BCG plus Mitomycin C vs. BCG				
Kaasinen 2003	+	-	+	2
Stasi 2006	++	-	+	3
Oosterlinck 2011	+	-	+	2
Gulpinar 2012	+	-	-	1
El Mohsen 2010	+	-	-	1
BCG plus Mitomycin C vs. Mitomycin C alone				
Rintala 1996	+	-	-	1
Jarvinen 2012	+	-	-	1
Witjes 1998	+	-	-	1
BCG plus Epirubicin vs. BCG alone				
Cai 2008	++	++	+	5
Bilen 2000	+	-	-	1
BCG plus Interferon α-2b vs. BCG alone				
Nepple 2010	++	-	-	2

Table S3 Quality assessment for retrospective study using Newcastle-Ottawa Scale

Studies	Selection	Comparability	Outcome/exposure	Total Score
Maintenance vs. Induction				
Andius 2004	★★★★		★★★	7
Okamura 2011	★★★★	★★	★★★	9
Decobert 2008	★★★★	★★	★★★	9
Low-dose vs. Standard-dose				
Takashi 1995	★★★★	★★	★★★	9
Yoneyama 2008	★★★★	★★	★★★	9
Irie 2003	★★★★	★★	★★★	9
BCG plus mitomycin C vs. BCG				
Badalato 2011	★★★★	★	★★★	8
BCG plus Epirubicin vs. BCG alone				
Tozawa 2001	★★★★	★★	★★★	9
BCG plus Interferon α-2b vs. BCG alone				
Bazarbashi 2000	★	★	★	3

Fig. S1 Summary of cessation-free survival for combination of BCG and Mitomycin C (A, BCG plus Mitomycin C vs. BCG alone; B, BCG plus Mitomycin C vs. Mitomycin C alone)

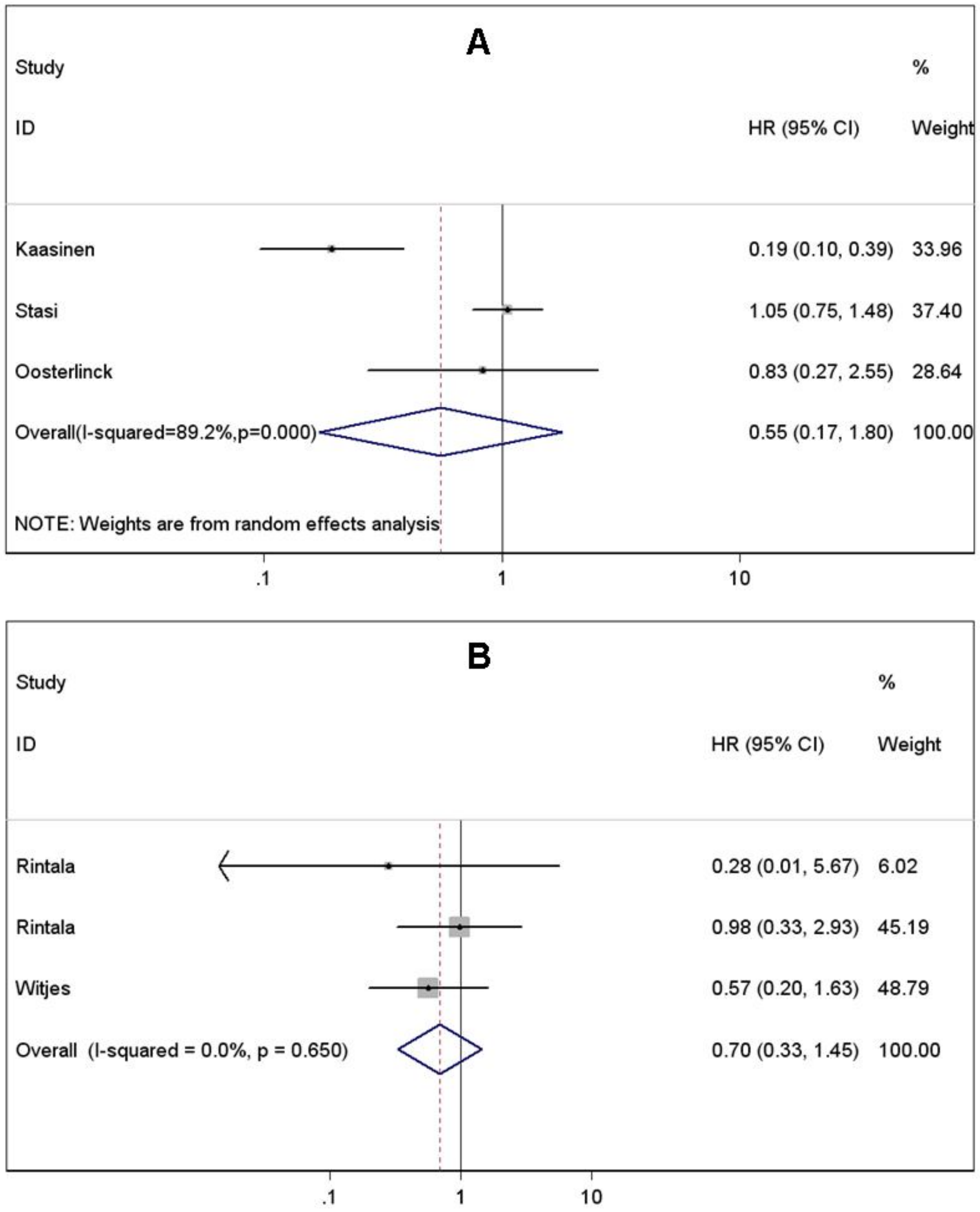

Fig. S2 Summary risk difference of side-effect for various BCG therapy schedules and doses

Study	Experiment Events	Experiment Total	Control Events	Control Total	Weight	Risk Difference 95% CI
**Bladder Contracture**						
BCG plus Epirubicin vs. BCG alone						
Bilen 2000	1	20	0	21	38.7%	0.05 [-0.08, 0.18]
Tozawa 2001	0	24	2	50	61.3%	-0.04 [-0.12, 0.04]
Total (95% CI)		44		71	100.0%	-0.01 [-0.08, 0.07]
**Epididymitis/Prostatitis/Urethral Infections**						
BCG maintenance vs. Induction						
Hinotsu 2011	1	42	0	42	100.0%	0.02 [-0.04, 0.09]
Total (95% CI)		42		42	100.0%	0.02 [-0.04, 0.09]
BCG Low-dose vs. Standard-dose						
Yalcinkaya 1998	1	25	2	25	100.0%	-0.04 [-0.17, 0.09]
Total (95% CI)		25		25	100.0%	-0.04 [-0.17, 0.09]
**Hematuria**						
BCG maintenance vs. Induction						
Decobert 2008	39	42	30	42	54.6%	0.21 [0.06, 0.37]
Hinotsu 2011	17	31	7	40	45.4%	0.37 [0.16, 0.58]
Total (95% CI)		73		82	100.0%	0.29 [0.16, 0.42]
BCG Low-dose vs. Standard-dose						
Yoneyama 2008	9	65	21	85	37.9%	-0.11 [-0.23, 0.02]
Yalcinkaya 1998	14	25	6	25	28.1%	0.32 [0.06, 0.58]
Irie 2003	3	34	7	30	34.0%	-0.15 [-0.32, 0.03]
Total (95% CI)		124		140	100.0%	-0.00 [-0.24, 0.23]
BCG plus Epirubicin vs. BCG alone						
Bilen 2000	4	20	8	21	100.0%	-0.18 [-0.45, 0.09]
Total (95% CI)		20		21	100.0%	-0.18 [-0.45, 0.09]
**LUTS**						
BCG maintenance vs. Induction						
Hinotsu 2011	39	42	30	42	54.6%	0.21 [0.06, 0.37]
Decobert 2008	17	31	7	40	45.4%	0.37 [0.16, 0.58]
Total (95% CI)		73		82	100.0%	0.29 [0.16, 0.42]
BCG Low-dose vs. Standard-dose						
Yoneyama 2008	31	65	48	85	54.1%	-0.09 [-0.25, 0.07]
Yalcinkaya 1998	19	25	24	25	18.4%	-0.20 [-0.38, -0.02]
Irie 2003	10	37	20	38	27.5%	-0.26 [-0.47, -0.04]
Total (95% CI)		127		148	100.0%	-0.15 [-0.27, -0.04]
BCG plus Epirubicin vs. BCG alone						
Bilen 2000	7	20	9	21	38.7%	-0.08 [-0.38, 0.22]
Tozawa 2001	13	24	20	50	61.3%	0.14 [-0.10, 0.38]
Total (95% CI)		44		71	100.0%	0.06 [-0.13, 0.24]
**Fever/Chills/Flu Symptoms**						
BCG maintenance vs. Induction						
Hinotsu 2011	18	42	11	42	54.6%	0.17 [-0.03, 0.37]
Decobert 2008	7	31	2	40	45.4%	0.18 [0.01, 0.34]
Total (95% CI)		73		82	100.0%	0.17 [0.04, 0.30]
BCG Low-dose vs. Standard-dose						
Yoneyama 2008	12	65	20	85	54.3%	-0.05 [-0.18, 0.08]
Yalcinkaya 1998	10	25	12	25	18.4%	-0.08 [-0.35, 0.19]
Irie 2003	2	35	5	39	27.2%	-0.07 [-0.20, 0.06]
Total (95% CI)		125		149	100.0%	-0.06 [-0.16, 0.03]
BCG plus Epirubicin vs. BCG alone						
Bilen 2000	2	20	3	21	38.7%	-0.04 [-0.24, 0.16]
Tozawa 2001	5	24	7	50	61.3%	0.07 [-0.12, 0.26]
Total (95% CI)		44		71	100.0%	0.03 [-0.11, 0.16]
BCG plus Epirubicin vs. BCG alone						
Nepple 2010	38	346	16	324	100.0%	0.06 [0.02, 0.10]
Total (95% CI)		346		324	100.0%	0.06 [0.02, 0.10]

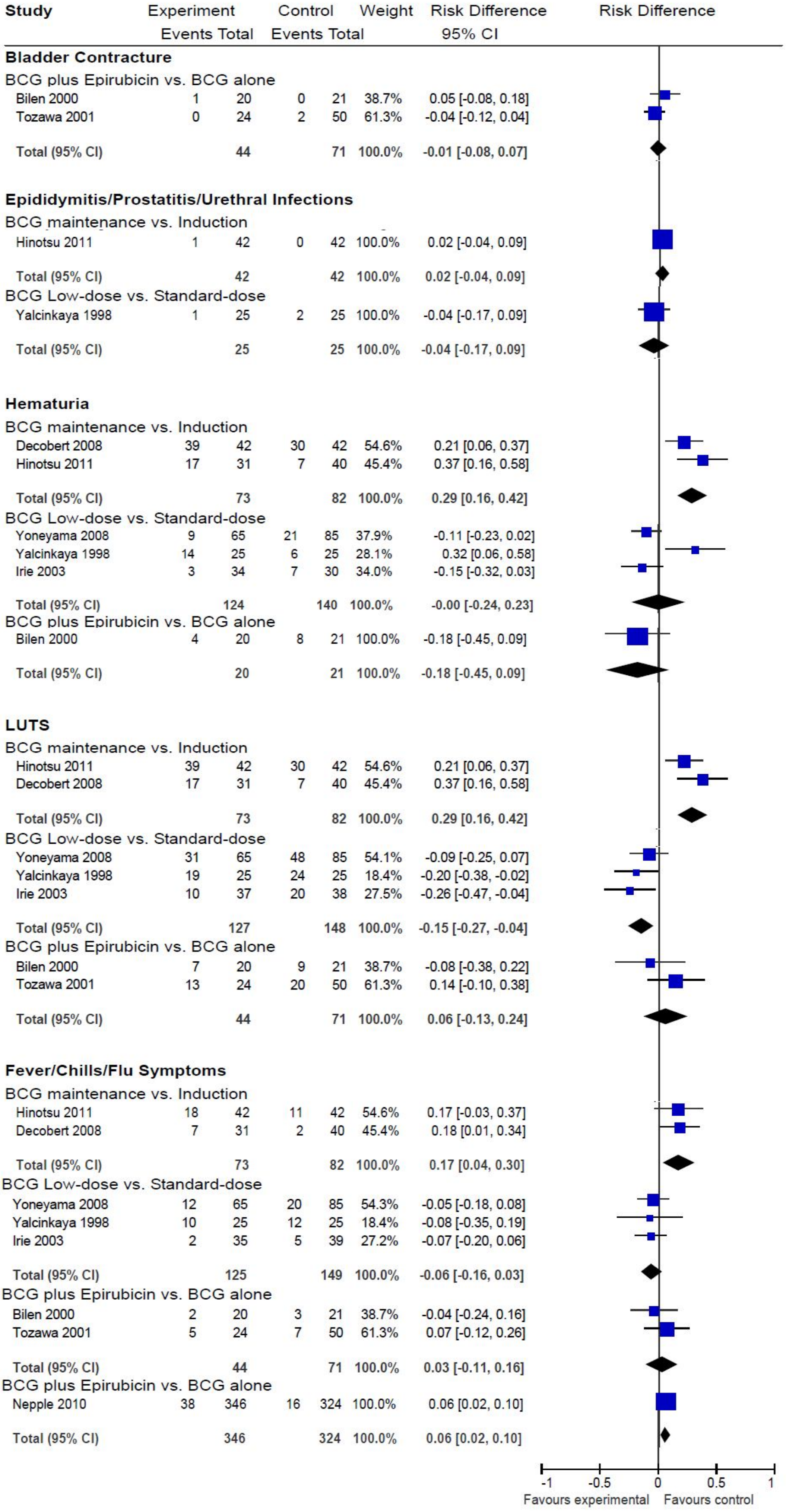

Supplement: Additional file 1: Table S1 — Side effects of various BCG therapy schedules and doses. Table S2. Quality assessment for RCTs using Jadad (5-ponit). Table S3. Quality assessment for retrospective study using Newcastle-Ottawa Scale. Figure S1. Summary of cessation-free survival for combination of BCG and Mitomycin C (A, BCG plus Mitomycin C vs. BCG alone; B, BCG plus Mitomycin C vs. Mitomycin C alone). Figure S2. Summary risk difference of side-effect for various BCG therapy schedules and doses. [file 1471-2407-13-332-S1.pdf]
